# 

**Published:** 1885-08-20

**Authors:** 


**☞ Notice.**--- Subscribers in arrears are requested to remit their dues at once. Please take due notice and govern yourselves accordingly.

### EDITORIAL NOTICES.

AN AGED DOCTOR.—Dr. C. C. Graham, who recently died in Louisville, Ky., had attained the advanced age of one hundred years and six months.

COCAINE is claimed to be curative to both the opium and alcohol habits. The method advised for its use is to hypodermically inject one-fourth or one-half grain of the muriate, dissolved in water, as often as the occasion may demand.

☞RECEIPTS will appear in our next.

